# Supplementary material for: The community as an active part in the implementation of interventions for the prevention and care of tuberculosis: A scoping review
Source: PLOS Glob Public Health. 2023 Dec 15;3(12):e0001482. doi: 10.1371/journal.pgph.0001482 (PMC10723726; doi:10.1371/journal.pgph.0001482)
Supplement: S2 File — (DOCX) [file pgph.0001482.s002.docx]

**S2 File**. Protocol

**Protocol: The community as an active part in the implementation of interventions for the prevention and care of tuberculosis: a scoping review protocol**

Community-led interventions include a variety of approaches in which communities take an active role in improving their health. We will identify and evaluate studies in which the community has actively participated to strengthen tuberculosis control programs.

**Research questions:**

**Primary question:**

¿What is known about tuberculosis control interventions and programs in which the community has been an active part in the development and implementation of the study?

**Secondary questions:**

a) ¿What strategies are being used to strengthen the participation of communities in interventions and programs for tuberculosis control?

b) ¿What opportunities, lessons learned, and challenges impact the sustainability of community-driven interventions and programs?

**Community stakeholders:** local government, national TB programs, Ministries of Health, Non-Governmental Organizations (NGOs).

**Methodology:**

**STEP 1: Collection and identification of studies**

To identify potential studies, we will first select search terms similar to "Community-Based related", "Community-based interventions", "participatory action research", "participatory engagement", "Community Participation", and "tuberculosis".

**Inclusion criteria:**

- We will consider all types of studies published in English and Spanish (randomized controlled trials, non-randomized trials, and observational studies). There will be no time restriction.
- We will consider studies in which the community has actively participated in the development and implementation of tuberculosis control programs.

**Exclusion criteria:**

- We will exclude editorials, expert opinions, review articles, book reviews, or conference abstracts, as well as traditional studies in which there is no involvement of the community in any phase of the research or tuberculosis control programs.

**The following databases will be searched:**

1. Medline (Pubmed)
2. Scopus
3. ERIC
4. Global Index Medicus
5. Scielo
6. LILACS

**Gray literature search:**

- Google Scholar (first 200 results)
- MedRxiv

**Manual search:**

- Search in recognized/highly relevant scientific journals.

1. Bulletin of the International Union Against Tuberculosis and Lung Disease
2. Eastern Mediterranean Health Journal
3. Infectious Diseases of Poverty
4. Global Public Health
5. African Health Sciences
6. Tubercle and Lung Disease
7. American Journal of Tropical Medicine and Hygiene
8. The International Journal of Tuberculosis and Lung Disease
9. African Health Sciences
10. Social Science & Medicine
11. PLoS One
12. The Lancet Infectious Diseases
13. Revista Peruana de Medicina Experimental y Salud Pública
14. Global Health: Science and Practice
15. Community Development Journal
16. Revista de Saúde Pública
17. Journal of Epidemiology and Community Health
18. African Journal of Primary Health Care & Family Medicine
19. Nigerian Journal of Medicine
20. Eastern Mediterranean Health Journal
21. Canadian Journal of Public Health
22. International Journal of Public Health Research
23. International Journal of Environmental Research and Public Health
24. Journal of Health Organization and Management
25. BMC Public Health
26. Public Health
27. Journal of Health Communication

- Detailed search in the bibliographical references section of selected highly relevant studies/articles.

**STEP 2: Selection of studies**

- Identification of studies according to inclusion criteria in the databases and manual search.
- Removal of duplicates.
- Sequential evaluation of titles and abstracts.
- Full-text evaluation.

One reviewer (LCR) will conduct the initial identification of titles and removal of duplicates using a reference manager (EndNote X9). Before starting the study selection process, a calibration process will be conducted between two reviewers (LCR and MB) using 10 randomly selected studies to ensure consistent selection of studies based on eligibility criteria.

**STEP 3: Extraction/encoding**

- For data extraction, the "descriptive-analytic" method will be employed, which involves applying a common analytical framework to all included studies and collecting standard information from each study. In addition, for information coding, a full-text evaluation of the included articles will be conducted based on the research questions. "Community involvement in research" and "strengths of community participation" will be extracted from the methods section of scientific articles, and the evaluation of "intervention sustainability" will be taken from the discussion section of the studies.

**STEP 4: Data analysis/presentation**

- The results will be presented through diagram tables that summarize the different modalities of community members' participation in the different aspects evaluated such as promotion and prevention of tuberculosis (health education, BCG vaccination and/or administration of chemoprophylaxis), detection and active case finding, treatment outcomes and adherence to anti-tuberculosis treatment (identification of barriers and difficulties for anti-tuberculosis treatment/supervision and monitoring during anti-tuberculosis treatment), implementation of interventions for tuberculosis control, and social support programs for tuberculosis-affected patients. In addition, evidence on how the community has been strengthened in their participation will be synthesized, and finally, the challenges in implementing these interventions (sustainability and cost-effectiveness studies) will be discussed.
