# Supplementary material for: The community as an active part in the implementation of interventions for the prevention and care of tuberculosis: A scoping review
Source: PLOS Glob Public Health. 2023 Dec 15;3(12):e0001482. doi: 10.1371/journal.pgph.0001482 (PMC10723726; doi:10.1371/journal.pgph.0001482)
Supplement: S6 File — (DOCX) [file pgph.0001482.s006.docx]

**Título completo: La comunidad como parte activa en la implementación de intervenciones para la prevención y atención de la tuberculosis: una revisión sistemática exploratoria**

**Título corto: Involucrar a la comunidad en la prevención y atención de la tuberculosis: una revisión sistemática exploratoria**

**Autores**

Lesly Chávez-Rimache^1^, César Ugarte-Gil^1,2*^, María J Brunette^2,3^

**Afiliaciones**

^1^ Instituto de Medicina Tropical Alexander von Humboldt, Universidad Peruana Cayetano Heredia, Perú

^2^ Facultad de Medicina, Universidad Peruana Cayetano Heredia, Perú

^3^ School of Health & Rehabilitation Sciences, College of Medicine. The Ohio State University, Estados Unidos de Norteamerica

[*cesar.ugarte@upch.pe](mailto:*cesar.ugarte@upch.pe)

**Resumen**

Las intervenciones que involucran a miembros de la comunidad incluyen una variedad de enfoques en los que dichos miembros desempeñan un papel activo para mejorar su salud. Evaluamos estudios en los que la comunidad ha participado activamente para fortalecer los programas de prevención y atención de la tuberculosis. Se realizó una búsqueda bibliográfica en Pubmed, Scopus, ERIC, Global Index Medicus, Scielo, Cochrane Library, LILACS, Google Scholar, revistas especializadas y otras referencias bibliográficas. La pregunta principal para esta revisión fue: ¿qué se sabe sobre las intervenciones y programas de control de la tuberculosis en los que la comunidad ha sido parte activa?

Dos revisores realizaron la búsqueda, el tamizaje y la selección de los estudios de forma independiente. En los casos de discrepancias sobre la elegibilidad de un artículo, se resolvió por consenso. Se seleccionaron 130 estudios, de los cuales el 68,47% (n=89/130) fueron publicados después de 2010. Los estudios se realizaron en África (44,62%), América (22,31%) y el sudeste asiático (19,23%). Se encontró que el 20% (n=26/130) de los estudios evaluaron la participación de la comunidad en la detección/búsqueda activa de casos, el 20,77% (n=27/130) en la promoción/prevención de la tuberculosis; 23,07% (n=30/130) en identificación de barreras al tratamiento, 46,15% (n=60/130) en supervisión durante el tratamiento y 3,08% (n=4/130) en apoyo social al paciente. La participación comunitaria no sólo fortalece las capacidades de los sistemas de salud para la prevención y atención de la tuberculosis, sino que también permite una mejor comprensión de la enfermedad desde la perspectiva del paciente y la comunidad afectada al identificar barreras y dificultades a través de la cascada de atención de la tuberculosis. Involucrar a los miembros de la comunidad en la creación conjunta de soluciones ofrece un camino fundamental para que los gobiernos locales erradiquen la tuberculosis.

**Introducción**

La tuberculosis (TB) es una enfermedad infecciosa que, a pesar de ser prevenible y curable, sigue siendo un problema de salud pública mundial, habiéndose estimado que 1,6 millones de personas murieron a causa de esta enfermedad en 2021 [1]. Además, acabar con la TB se ha convertido en un desafío mayor al estar relacionada con otras enfermedades como el virus de la inmunodeficiencia humana (VIH). En la epidemiología de la tuberculosis, los determinantes sociales de la salud en una comunidad como la pobreza, el hacinamiento, las condiciones inadecuadas de vivienda, la desnutrición, etc. ejercen una clara influencia en todas las etapas de la tuberculosis (riesgo de exposición, tiempo hasta el diagnóstico, tratamiento, susceptibilidad a progresión de la enfermedad y en la retención en el cuidado) [2, 3]. Por tanto, la tuberculosis es una enfermedad social que requiere del involucramiento de la comunidad para proponer soluciones conjuntas con el gobierno local [4].

Para acabar con la TB, una de las estrategias utilizadas es la terapia acortada directamente observada (DOTS) [5]. Esta estrategia ha sido administrada inicialmente bajo la supervisión guiada de trabajadores de la salud, pero a lo largo de los años también ha incluido voluntarios de la comunidad y miembros de las familias de los pacientes. Esto ha llevado a incluir paulatinamente a la comunidad en las actividades de prevención y atención de la tuberculosis. Esta participación comunitaria ha ido escalando a otros niveles como la detección y búsqueda activa de personas con síntomas de TB, y esta participación ayuda a generar una mayor vinculación de los pacientes con los centros de atención de TB y a aumentar la retención en la atención en el cuidado de los casos detectados [6].

A lo largo de los años, la participación comunitaria en programas destinados a prevenir y eliminar la tuberculosis ha logrado avances significativos, lo que les ha permitido integrarse en planes estratégicos como el DOTS, Alto a la Tuberculosis y Fin a la Tuberculosis [7-10]. El enfoque de la Organización Mundial de la Salud (OMS), llamado “ENGAGE-TB”, enfatiza que la participación comunitaria es fundamental para mejorar el alcance y la sostenibilidad de los servicios de tuberculosis para la comunidad. Este enfoque permite la implementación de actividades comunitarias integradas contra la tuberculosis dentro de los programas de salud, y la OMS proporciona orientación técnica, capacitación comunitaria y fomenta la creación y el desarrollo de alianzas entre los programas de atención de la tuberculosis y las sociedades civiles [11, 12]. Además, el quinto componente de la Estrategia Alto a la Tuberculosis enfatiza la importancia de la comunicación, la promoción y la movilización social para mejorar la detección de casos, la adherencia al tratamiento, empoderar a las personas afectadas por la tuberculosis, combatir el estigma y la discriminación, movilizar el compromiso político y asignar recursos para la prevención de la tuberculosis. y cuidado [7]. Además, se fomenta la participación de la comunidad en la investigación, contribuyendo así al fortalecimiento de los programas de prevención y atención de la tuberculosis. Un ejemplo de este esfuerzo es TB Alliance, que es una asociación que trabaja con personas afectadas por la TB, a quienes capacita y empodera para mejorar sus conocimientos y habilidades para participar en la investigación de medicamentos contra la tuberculosis mediante la creación de una extensa red de Consejos de Asesores Comunitarios (CAC). Esta iniciativa de extensión comunitaria permite crear vínculos entre los participantes de ensayos clínicos, miembros de la comunidad e investigadores académicos para lograr una retroalimentación mutua basada en un diálogo abierto y consensuado [13].

La investigación participativa basada en la comunidad (CBPR las siglas en inglés) es un enfoque en el que la comunidad participa en todas las etapas de la investigación desde la concepción, el diseño, la ejecución, la implementación y el seguimiento de la investigación [14]. La CBPR permite la creación de asociaciones colaborativas e igualitarias entre miembros de la comunidad e investigadores académicos. Además, este enfoque de investigación surgió con la necesidad de reducir las brechas comunicativas y operativas que frecuentemente implican un fracaso para la investigación tradicional que muchas veces omite las complejas interrelaciones culturales, sociales y económicas y su impacto en la implementación efectiva de las intervenciones [14-17]. La CBPR se puede utilizar para todos los diseños de estudios, desde estudios cualitativos hasta ensayos clínicos aleatorios [14]. Hasta donde sabemos, sólo hay dos revisiones de Arshad et al.[6] y Musa et al. [18], quienes evaluaron el efecto de las intervenciones comunitarias para la prevención y la atención de la tuberculosis y reportaron beneficios potenciales al incluir a la comunidad en los estudios de investigación sobre la tuberculosis. Sin embargo, actualmente se desconoce hasta qué punto la participación comunitaria puede generar beneficios para los programas de salud enfocados en la TB que terminan considerando los principios de CBPR (reconocer a la comunidad como una unidad de identidad, aprovechar las fortalezas y recursos de la comunidad, facilitar alianzas colaborativas, integrar conocimientos y acciones para el beneficio mutuo de todos, promover un proceso de co-aprendizaje y empoderamiento comunitario, emprender un proceso cíclico e iterativo, abordando la salud desde un enfoque integral y ecológico, y difundiendo los hallazgos y conocimientos adquiridos a todos los socios). Asimismo, se desconoce cuáles han sido los mecanismos que se han utilizado para fortalecer a las comunidades en su participación y cuál ha sido el impacto de la inclusión de la comunidad para la prevención y atención de la tuberculosis. Con base en esto, nuestra revisión pretende evaluar las características de las intervenciones en las que la comunidad ha tenido una participación genuinamente activa en el desarrollo e implementación de estudios de investigación para fortalecer los programas de prevención y control de la tuberculosis.

**Métodos**

**Diseño del estudio**

Llevamos a cabo una revisión sistemática exploratoria siguiendo las pautas de los Elementos de informe preferidos para revisiones sistemáticas y la extensión de metaanálisis para revisiones de alcance (PRISMA-ScR) [19] (Archivo S1) y un protocolo que se llevó a cabo a priori, basado en lo siguiente preguntas de investigación:

*Pregunta principal: ¿Qué se sabe sobre las intervenciones y programas de control de la tuberculosis en los que la comunidad ha sido parte activa en el desarrollo e implementación del estudio?*

*Preguntas secundarias: a) ¿Qué estrategias se están utilizando para fortalecer la participación de las comunidades en las intervenciones y programas de control de la tuberculosis? y b) ¿Qué oportunidades, lecciones aprendidas y desafíos existen en la sostenibilidad de las intervenciones y programas impulsados por la comunidad?*

El protocolo está disponible en el Archivo S2. Esta revisión del alcance siguió el marco metodológico de cinco etapas desarrollado por Arksey y O'Malley [20] y una sexta etapa adicional desarrollada por Levac et al. [21], estas etapas son las siguientes: 1) identificar la pregunta de investigación, 2) identificar estudios relevantes, 3) seleccionar estudios, 4) registrar datos, 5) cotejar, resumir e informar resultados, y 6) consultar con las partes interesadas relevantes.

**Fuentes de datos y búsqueda**

Para identificar los estudios, realizamos búsquedas sistemáticas en Medline (a través de Pubmed), Scopus, ERIC (Education Resources Information Center), Global Index Medicus, Scielo, Cochrane Library y LILACS (Literatura Latinoamericana y del Caribe en Ciencias de la Salud). Se realizó una búsqueda de literatura gris a través de Google Scholar y MedRxiv. Además, realizamos una búsqueda manual identificando las revistas de organizaciones internacionalmente reconocidas en tuberculosis (Archivo S3) y revisando las referencias bibliográficas de los artículos de revisión y estudios incluidos. Se utilizaron términos de búsqueda como “Relacionados con la comunidad”, “Intervenciones basadas en la comunidad”, “investigación acción participativa”, “participación participativa”, “Participación comunitaria” y “tuberculosis” (la lista completa de términos de búsqueda se puede encontrar en el archivo S4). La elección de términos y estrategias de búsqueda se desarrolló y perfeccionó mediante una discusión con el equipo de investigación (Archivo S5). Todas las búsquedas se realizaron hasta el 15 de julio de 2023 y se actualizó la búsqueda en Medline (a través de Pubmed) hasta el 30 de abril de 2022. Para la importación de artículos y eliminación de duplicados, el gestor de referencias utilizado fue Endnote X9 (Clarivate).

**Criteria de selección**

Esta revisión de alcance incluyó estudios que describen o analizan el papel de la participación directa de miembros clave de la comunidad en el desarrollo e implementación de programas de prevención y control de la tuberculosis. Se incluyeron estudios como ensayos clínicos aleatorios, ensayos no aleatorios, estudios observacionales y *preprints* en los que la comunidad participó activamente en un estudio de investigación o programas enfocados en la prevención, diagnóstico, tratamiento y eliminación de la tuberculosis (planificación de la idea, diseño, implementación, seguimiento y evaluación de las intervenciones). Se realizó una búsqueda de estudios publicados de todos los tiempos en español e inglés. Se excluyeron editoriales, opiniones de expertos, artículos de revisión, reseñas de libros o resúmenes de congresos y estudios tradicionales en los que la comunidad solo se consideraba un medio para responder al objetivo del estudio. La Fig. 1 incluye los procesos de identificación y selección de estudios según los criterios de elegibilidad.

**Selección, recopilación y procesamiento de datos.**

Un revisor (LCR) realizó la identificación inicial del título y la eliminación de duplicados utilizando un administrador de referencias Endnote X9 (Clarivate). Antes de iniciar el proceso de selección de estudios, se realizó un proceso de calibración entre dos revisores (LCR y MJB) con 10 estudios seleccionados aleatoriamente para seleccionar los estudios según los criterios de elegibilidad. Posteriormente, en una primera fase se realizó una selección de los estudios con base en los títulos y resúmenes; y en una siguiente fase se realizó una evaluación del texto completo según los criterios de elegibilidad. Todos estos procesos fueron supervisados de cerca por un investigador (MJB).

Para la extracción de datos se utilizó el método “descriptivo-analítico”, que consiste en aplicar un marco analítico común a todos los estudios incluidos y recopilar información estándar de cada estudio [20]. Además, para realizar la codificación de la información se realizó una evaluación de texto completo de los artículos incluidos a partir de las preguntas de investigación. La "inclusión de la comunidad en la investigación" y las "fortalezas de la participación comunitaria" se extraerán de la sección de métodos de los artículos científicos y la evaluación de la "sostenibilidad de las intervenciones" de la sección de discusión de los estudios primarios. Los resultados se presentaron en diagramas tabulares. Para codificar el nivel de participación comunitaria en las intervenciones, nos remitimos a la revisión sistemática realizada por AS George *et al*.[22]

En esta revisión, la participación comunitaria se evaluó con base en cinco elementos clave: identificación y definición de los problemas abordados, intervenciones para abordar estos problemas, implementación de intervenciones, gestión de recursos y seguimiento/evaluación de las intervenciones. Además, resumimos la evidencia de las diferentes modalidades de participación de los miembros de la comunidad en los diferentes aspectos evaluados de la cascada de atención de la tuberculosis pulmonar, como en la prevención de la tuberculosis (educación para la salud, vacunación BCG y/o administración de quimioprofilaxis), detección y búsqueda activa de casos, resultados y adherencia al tratamiento de la tuberculosis (identificación de barreras y dificultades para el tratamiento de la tuberculosis/supervisión y vigilancia durante el tratamiento de la tuberculosis), implementación de intervenciones para poner fin a la tuberculosis y programas de apoyo y apoyo social para pacientes afectados por la tuberculosis. Para ser incluidos en esta revisión de alcance, los artículos debían involucrar la participación de la comunidad en al menos una de las etapas de la cascada de atención de la tuberculosis. Respecto a las preguntas secundarias, codificamos información sobre cómo la comunidad se ha fortalecido en su participación en estudios o programas enfocados a la prevención y atención de la tuberculosis; y finalmente, codificamos información de estudios que evaluaron la sostenibilidad de intervenciones o programas (intervención o programa que continúa implementándose después de un período de tiempo definido mientras se adapta).

Además, evaluamos aquellos estudios que al involucrar a la comunidad cumplieron con todos los principios de CBPR. La investigación con metodología CBPR se basa en los principios de participación comunitaria participativa y equitativa en todos los procesos de investigación y propiedad compartida de la identificación de problemas, el desarrollo y los productos de investigación [17, 23-25]. Los principios fundamentales del enfoque CBPR son los siguientes: reconocimiento de la comunidad como una identidad única, se basa en las fortalezas y recursos de la propia comunidad, promueve el trabajo en equipo colaborativo/equitativo, facilita el aprendizaje mutuo y el desarrollo de capacidades a través de un proceso de empoderamiento continuo, integra y establece un equilibrio entre la generación de conocimiento y la acción, se utiliza un proceso cíclico e iterativo, permite la evaluación abordando los problemas de salud en la comunidad con un enfoque integral y ecológico, difunde los resultados a todos: academia, gobiernos locales, Organizaciones No-Gubernamentales (ONG) y miembros de la comunidad. Estos principios se adaptan al contexto sociocultural de las comunidades [14, 15].

**Resultados**

**Búsqueda de literatura**

Esta revisión identificó inicialmente 2077 títulos y resúmenes. De estos estudios, se excluyeron 1624 estudios según los criterios de elegibilidad. Se evaluaron 453 estudios a texto completo, de los cuales 175 estudios finalmente cumplieron con los criterios de inclusión preestablecidos. El diagrama de flujo de selección de estudios se presenta en la Fig. 1.

**Características de los artículos incluidos**

De los estudios incluidos, a lo largo de los años, ha habido un aumento gradual en la cantidad de investigaciones en las que la comunidad ha participado activamente para acabar con la tuberculosis. Los estudios identificados en nuestra revisión se realizaron en todas las regiones de la OMS, principalmente en la región de África (46,3%, n=81/175), las Américas (17,7%, n=31/175) y el Sudeste Asiático (20,0%, n=35/175). En relación a los tipos de artículos, los más frecuentes fueron los cuantitativos (45,7%, n=80/175) seguidos de los cualitativos (33,1%, n=58/175) y finalmente los métodos mixtos (14,3%, n =25/175). Estas características se presentan en la Tabla 1.

**Tabla 1. Características de los estudios incluidos en la revisión sistemática exploratoria. (n=175)**

| Características de los estudios | Total de los estudios=130  Numero (%) |
| --- | --- |
| Año de publicación |  |
| 1990-1999 | 7 (4.0) |
| 2000-2009 | 34 (19.4) |
| 2010-2021 | 134 (76.6) |
|  |  |
| Región |  |
| Africa | 81 (46.3) |
| Americas | 31 (17.7) |
| Sudeste Asiático | 35 (20.0) |
| Europa | 3 (1.7) |
| Mediterraneo oriental | 8 (4.6) |
| Pacifico Occidental | 16 (9.1) |
| Artico | 1 (0.6) |
|  |  |
| Diseño del estudio |  |
| Transversal | 9 (5.1) |
| Caso de estudio | 3 (1.8) |
| Cualitativo | 58 (33.1) |
| Métodos mixtos | 25 (14.3) |
| Estudios cuantitativos | 80 (45.7) |
|  |  |
| Idioma |  |
| Ingles | 172 (98.3) |
| Español | 3 (1.7) |

En relación con las preguntas planteadas en esta revisión del alcance, dividimos los resultados en tres secciones. El primero tiene que ver con la naturaleza de la participación comunitaria y el involucramiento comunitario en la implementación de intervenciones dirigidas a las diferentes etapas de la cascada de atención de la tuberculosis. La segunda sección trata sobre las estrategias que se han empleado para fortalecer la participación comunitaria en investigaciones y/o programas enfocados a la eliminación de la tuberculosis, y la tercera sección trata sobre la sostenibilidad de intervenciones en las que la comunidad ha jugado un papel activo en su desarrollo y costo. -estudios de eficacia de estas intervenciones.

**Naturaleza de la participación comunitaria activa en los estudios incluidos**

La participación de las comunidades en estudios de investigación sobre tuberculosis ha sido evaluada y categorizada considerando la clasificación del estudio de AS George *et al*.[22], quienes evaluaron el grado de participación comunitaria en investigaciones sobre intervenciones en sistemas de salud en países de bajos y medianos niveles. países de ingresos a través de cinco elementos diferentes: (1) identificación de los problemas abordados; (2) identificación y definición de intervenciones; (3) implementación de las intervenciones; (4) gestión de recursos para las intervenciones y (5) seguimiento y evaluación de las intervenciones. Sólo 4/130 (3,08%) estudios involucraron a la comunidad a través de estos cinco elementos. Los detalles de la naturaleza de la participación a través de estos cinco elementos se presentan en la Tabla 2.

**Tabla 2. Naturaleza de la participación comunitaria en los estudios incluidos.**

| Naturaleza de la participación comunitaria (PC) | Articulos con Activa Participación Comunitaria (APC) (n=175)  Numero (%) |
| --- | --- |
| Identificación de necesidades, factores de riesgo y/o definición de problemas | 111 (63.4) |
| Identificación y definición de intervenciones | 65 (37.1) |
| Implementación de intervenciones | 91 (52.0) |
| Gestión de recursos para la intervención | 44 (25.1) |
| Seguimiento, evaluación de intervenciones | 59 (33.7) |

APC: Activa Participación Comunitaria

**Participación comunitaria activa en la implementación de intervenciones**

En los estudios incluidos, la participación comunitaria en el desarrollo de estudios de investigación en las diferentes etapas de la cascada de atención de la tuberculosis o como parte de un programa de prevención y atención de la tuberculosis se identificó en los siguientes escenarios: 28,0% (n=49/175 ) de los estudios incluyeron a la comunidad en actividades relacionadas con la prevención de la tuberculosis, el 29,1% (n=51/175) de los estudios involucraron a la comunidad en la detección y búsqueda activa de casos de tuberculosis. El 24,0% (n=42/175) de los estudios en identificación de barreras y dificultades para el tratamiento antituberculoso, el 44,0% (n=77/175) de los estudios sobre supervisión y vigilancia del tratamiento antituberculoso y el 4,0% (n=77/175) de los estudios sobre supervisión y vigilancia del tratamiento antituberculoso 7/175) de los estudios evaluaron la participación de la comunidad en el apoyo social y el apoyo al paciente afectado por tuberculosis. Finalmente, se identificó que el 13,1% (n=23/175) de los estudios evaluaron la participación de la comunidad en otros aspectos relacionados como mayor comprensión de la enfermedad, identificación de factores relacionados con la búsqueda y calidad de la atención a los pacientes afectados. por la tuberculosis y estudios que exploraron la experiencia de los trabajadores de salud comunitarios para erradicar la tuberculosis (Tabla 3).

Por otro lado, con el paso de los años, uno de los factores que posiblemente incrementó la participación comunitaria fue el manejo conjunto de VIH-TB. Se encontró que el 16,6% (n=29/175) de los estudios evaluaron esta planificación conjunta de actividades VIH-TB. La mayoría de estos estudios se realizaron en África (n=21/29), seguida de América (n=4/29) y el sudeste asiático (n=2/29). Sólo se informó un estudio que evaluó el manejo y la planificación conjuntos para el manejo de la diabetes y la tuberculosis [26]. Por otro lado, el 32,6% (n=27/175) de los estudios se desarrollaron entre 2021 y 2023 (durante la pandemia de COVID-19), realizándose principalmente en África (42,1%) seguida del Sudeste Asiático (19,3%). y Pacífico Occidental (11,0%).

Tabla 3. Participación comunitaria activa en la implementación de intervenciones dirigidas a las diferentes etapas de la cascada de atención de la tuberculosis.

|  | Artículos con Participación Comunitaria (n=175)  Número (%) |
| --- | --- |
| Prevención TB | 49 (28.0) |
| Detección y búsqueda activa de casos | 51 (29.1) |
| Identificación de barreras al tratamiento de la tuberculosis. | 42 (24.0) |
| Supervisión y vigilancia del tratamiento de la tuberculosis. | 77 (44.0) |
| Apoyo social y apoyo al paciente. | 7 (4.0) |
| Otros | 23 (13.1) |

ACP: Participación comunitaria activa.

*Estudios en los que ha participado la comunidad para comprender la enfermedad, factores asociados a la búsqueda de atención y experiencias de participación comunitaria.

En cuanto a las actividades realizadas por la comunidad, encontramos que realizaron tareas como asistentes de investigación en algunas de las diferentes etapas de las investigaciones, como reclutar participantes para la investigación, ejecutar la implementación de intervenciones (por ejemplo, evaluar algunos indicadores epidemiológicos como como la prevalencia de la tuberculosis en su comunidad, liderar grupos focales para identificar brechas, dificultades y oportunidades en la prevención y atención de la tuberculosis, realizar encuestas para evaluar el nivel de conocimiento sobre la tuberculosis en su comunidad, realizar actividades educativas sobre la prevención y atención de la tuberculosis, realizar búsqueda activa de casos, coadyuvar en la agilización de los procesos de diagnóstico oportuno de tuberculosis, evaluación de la adherencia al tratamiento antituberculoso y realización de tareas de seguimiento de los pacientes brindando apoyo social y emocional). Incluso han realizado tareas para capacitar a otros miembros de la comunidad para que se conviertan en futuros promotores de salud en su comunidad. Lo hicieron involucrando a la comunidad a través de líderes de opinión y organizaciones de base.

**Fortalecimiento de la participación comunitaria en los estudios incluidos**

El 5,1% (n=9/175) de los estudios incluidos en nuestra revisión utilizaron el enfoque de investigación participativa basada en la comunidad. Sin embargo, sólo la mitad (n=4/175) de estos estudios cumplieron con los principios CBPR. Por otro lado, el 32,6% (n=57/175) de los estudios realizaron algún tipo de educación o capacitación a los trabajadores comunitarios para el desempeño de sus funciones en los estudios de investigación sobre tuberculosis.

**Evaluaciones de sostenibilidad y rentabilidad de los estudios incluidos**

El 4,6% (n=8/175) de los estudios evaluaron la sostenibilidad de las intervenciones en las que había participado la comunidad. Además, el 2,31% (n=3/130) de los estudios incluidos evaluaron la rentabilidad de la participación comunitaria en la atención de la tuberculosis [27-29]. Khan y otros [27] informaron que el tratamiento autoadministrado era el más rentable (164 dólares por paciente curado). Sin embargo, tuvieron una tasa de curación del 62% en comparación con el DOTS administrado por trabajadores de salud comunitarios (TSC) ($172 por caso curado), que tuvo una tasa de curación del 67%. El DOTS supervisado por un familiar ($185 por paciente curado) tuvo una tasa de curación del 55% y finalmente el DOTS administrado en un centro de salud ($310 por paciente curado) tuvo una tasa de curación del 58%. Sinanovic y otros [28] informaron que, para los pacientes nuevos, el DOTS basado en la comunidad era más rentable ($726 por paciente tratado con éxito) que ningún tratamiento observado directamente ($1201 por paciente tratado con éxito). De manera similar, para los pacientes que retrataban, el DOTS comunitario fue más rentable ($1,419 por paciente tratado con éxito) que ningún tratamiento observado directamente ($2,058 por paciente tratado con éxito). Finalmente, Prado et al. [29] informaron que el costo por paciente tratado con DOTS supervisado por tutores fue de $398 y para DOTS supervisado por trabajadores de salud comunitarios fue de $548.

Todos los estudios incluidos en nuestra revisión (cuantitativos y cualitativos) mostraron efectos positivos de la participación comunitaria en las diferentes etapas de la prevención y atención de la tuberculosis. Además, los autores informaron que la participación comunitaria mejoró varios aspectos de la investigación, desde la planificación del estudio, la implementación de la intervención, la evaluación y el seguimiento de los participantes. Por ejemplo, se agilizó el reclutamiento de participantes, y con mayor precisión la recolección de datos sensibles, la adherencia de los participantes al estudio, entre otros. Esto se debe a que los pacientes tenían mayor confianza en los trabajadores de salud comunitarios. Si bien, efectivamente, los resultados aún no son concluyentes y se requieren más estudios adecuadamente diseñados y adaptados de acuerdo a los diferentes contextos socioculturales y económicos de cada país o región, la participación comunitaria ha sido y seguirá siendo parte del avance y fortalecimiento de los programas de prevención y atención de la tuberculosis.

**Discusión**

En nuestra revisión de alcance, identificamos 175 estudios que informaron una participación genuina de los miembros de la comunidad. La mayoría de estos estudios se realizaron en África (46,3%), la región de las Américas (17,7%) y Asia (20,0%). Según el Informe mundial sobre la tuberculosis de 2022, la mayor carga de enfermedad tuberculosa se produce en las regiones de Asia y África (69%) [30]. Esto podría explicar la razón de la mayor frecuencia de estudios encontrados en estos continentes. Este escenario es diferente para la región de las Américas, en la que su carga global de tuberculosis es del 2,9%, pero es la segunda región con más estudios que intentan involucrar a la comunidad como parte de sus estrategias para acabar con la tuberculosis. Sin embargo, la mayoría de estos estudios se llevan a cabo en poblaciones con una alta carga de tuberculosis, como en los países de América Latina y el Caribe [31].

Los resultados de nuestra revisión de alcance sugieren que la participación comunitaria activa contribuye a fortalecer los programas de prevención y atención de la tuberculosis. Encontramos que la comunidad participó principalmente en la identificación de necesidades, factores de riesgo y/o problemas (63,4%); y en la implementación de intervenciones (52,0%). Por otro lado, encontramos que la comunidad participó principalmente en la supervisión y seguimiento del tratamiento antituberculoso (44,0%), en la identificación de barreras al tratamiento (24,0%), en la prevención (28,0%) y en la búsqueda activa de casos (29,1%). Esto pone de relieve la necesidad de alejarse del escenario tradicional de la investigación científica, que está dirigida únicamente por investigadores académicos. En lugar de ello, deberíamos considerar un enfoque integral y ecológico que también involucre a la comunidad y otros actores sociales como actores clave en la identificación de problemas y el desarrollo de soluciones conjuntas que reduzcan las brechas sociales y económicas que contribuyen a la carga de la tuberculosis. Esto es similar a lo mencionado por Arshad et al.[6], quienes informaron que las intervenciones comunitarias aumentaron la probabilidad de detección de casos de tuberculosis (RR: 3,1; IC 95%: 2,92 a 3,28) y las tasas de éxito del tratamiento (RR : 1,09; IC 95%: 1,07 a 1,11). Asimismo, informaron que los trabajadores de salud comunitarios, al entregar el tratamiento, no sólo aumentaron y mejoraron las condiciones de acceso a la atención de los pacientes afectados por tuberculosis, sino que también mejoraron los sistemas de registro y notificación de casos de tuberculosis. Yassin y otros [32] implementaron un paquete de intervención comunitaria contra la tuberculosis para acercar los servicios de diagnóstico y tratamiento a las comunidades vulnerables y reportaron que la participación comunitaria duplicó las tasas de notificación de casos de tuberculosis y mejoró los resultados del tratamiento. De manera similar, otros estudios han informado que la búsqueda activa de casos en países o lugares con una alta carga de tuberculosis por parte de los trabajadores de salud comunitarios, voluntarios comunitarios y familiares capacitados podría fortalecer los sistemas de notificación y reducir las brechas en el acceso a la información y la atención médica en la comunidad. La búsqueda activa de casos bacilíferos contribuyó de manera importante al éxito del diagnóstico y tratamiento de la tuberculosis. Esto es importante porque los pacientes con tuberculosis frecuentemente acuden a los centros de salud cuando su enfermedad ha empeorado, y esto es una limitación importante en los esfuerzos globales para poner fin a la tuberculosis [33-43].

Con respecto a los resultados del tratamiento de la tuberculosis, se han informado mejores resultados cuando los trabajadores de salud comunitarios administran DOTS, lo que permite mayores tasas de éxito del tratamiento. Además, los trabajadores de salud comunitarios han contribuido a la supervisión y seguimiento de los casos de farmacorresistencia y al apoyo a los pacientes durante toda la fase de tratamiento. En cuanto a las estrategias para fortalecer la participación comunitaria, encontramos que solo 4 estudios involucraron a la comunidad en todas las fases de la investigación y permitieron un compromiso más estrecho entre la comunidad y los investigadores académicos [15, 44-4].

En los estudios con un enfoque CBPR, los investigadores académicos suelen establecer un consejo asesor comunitario (o CAB), que está formado por miembros de la comunidad que representan las voces de la comunidad con respecto a sus percepciones, preferencias, talentos, etc. y prioridades. Estos socios comunitarios pueden ser centros de salud comunitarios, departamentos de salud pública, escuelas, prisiones y organizaciones de la sociedad civil, como organizaciones vecinales [14, 47, 48]. Un caso de asociación colaborativa con enfoque CBPR es un estudio que realizamos en la población afectada por TB en el Perú en colaboración entre la Universidad Peruana Cayetano Heredia, Ohio State University y la Asociación de Pacientes Tuberculosos en Comas [ASET] (Comas, distrito del Perú con una alta tasa de tuberculosis) [49]. En la primera fase, llevamos a cabo un programa de empoderamiento de los trabajadores de salud comunitarios en ASET a través de capacitación en investigación, y en la segunda fase, implementamos una intervención liderada por los trabajadores de salud comunitarios para evaluar el efecto de los determinantes sociales de la salud en la tuberculosis en la población afectada por la tuberculosis.

La inclusión de alianzas estratégicas de investigación que permitan el establecimiento de una relación equitativa entre la comunidad y los investigadores académicos requiere tiempo y recursos financieros [50]. En los países de ingresos bajos y medios, los trabajadores de salud comunitarios son un componente clave del personal sanitario para alcanzar las Metas de Desarrollo Sostenible [51]. Sin embargo, los trabajadores de salud comunitarios enfrentan muchos desafíos, como una alta rotación en sus funciones, baja motivación, supervisión inadecuada, falta de recursos disponibles para que puedan llevar a cabo sus funciones, compensación o incentivos insuficientes y poco reconocimiento y participación por parte de los proveedores de atención médica. Todo esto limita su capacidad para contribuir eficazmente a la atención primaria de salud [52].

Entre las funciones informadas de trabajadores de salud comunitarios en los estudios incluidos se encuentran determinar indicadores epidemiológicos como la prevalencia de la tuberculosis en su comunidad, liderar grupos focales para identificar brechas, dificultades y oportunidades en la prevención y atención de la tuberculosis, brindar educación sanitaria (aumentar la conciencia y el conocimiento sobre la tuberculosis), detección y búsqueda activa de casos, apoyando procesos de diagnóstico oportuno, evaluando la adherencia al tratamiento y el seguimiento de los pacientes. Sin embargo, los trabajadores de salud comunitarios no sólo han participado en las funciones antes mencionadas, sino que a menudo han resuelto problemas que los programas de tuberculosis ignoran, como brindar comodidad moral personalizada, apoyo nutricional y reducción del estigma a los pacientes afectados por la tuberculosis [53]. Entre los factores que motivan a los ACS se encuentran la satisfacción por ayudar a las personas afectadas por la tuberculosis (sentimiento de prestigio relacionado con ayudar al prójimo), tener una buena relación con los trabajadores de la salud, el respeto por la comunidad y el beneficio personal que obtienen al aprender nueva información sobre la tuberculosis y salud general. Este último fue el principal factor de motivación para los trabajadores de salud comunitarios, ya que les empoderaba tanto a ellos como a los pacientes sobre su salud y la de su comunidad [54]. Además, los trabajadores de salud comunitarios pueden disminuir el estigma de la enfermedad de la tuberculosis porque, al aprovechar sus propias experiencias personales sobre la enfermedad, pueden aumentar la apertura y disminuir el estigma entre las personas [4, 55, 56].

Por otro lado, sólo el 32,6% de los estudios capacitaron a los trabajadores de salud comunitarios para que pudieran realizar sus funciones, las cuales fueron variadas, pero principalmente enfocadas a implementar intervenciones. Sin embargo, la mayoría de estos estudios solo los capacitaron para realizar una función específica dentro del estudio de investigación.

Reyes y otros [56] fue el único estudio incluido que llevó a cabo un programa de capacitación de promotores comunitarios para la prevención y atención de la tuberculosis en México. Este estudio empoderó a sus promotores a través de talleres educativos y participativos para mejorar la comprensión de la enfermedad y sobre la prevención y el tratamiento de la enfermedad.

Por otro lado, la participación activa de la comunidad se reflejó en su desempeño como asistentes de investigación (o participación en alguna fase del estudio) al desarrollar tareas como reclutar participantes, implementar intervenciones (brindar tratamientos, conducir grupos de profesores, etc.), recopilación de datos (por ejemplo muestreo, encuestas, etc.), seguimiento de los participantes, educación sanitaria a los pacientes, entre otros. Esto no sólo ayudó a agilizar los procesos de investigación, sino que también podría mejorar la precisión de la información recopilada y la validez de los resultados. Además, formar estos vínculos entre los investigadores y la comunidad mejora y restablece la confianza para futuras investigaciones.

En relación a los estudios de sostenibilidad y costo-efectividad de las intervenciones, la sostenibilidad de las intervenciones son aquellos programas que continúan implementándose después de un período de tiempo definido mientras se adaptan y adaptan para seguir produciendo beneficios para las personas [57, 58]. La sostenibilidad es un componente importante para la implementación y difusión de intervenciones de salud y para la evaluación de sus efectos en el mediano y largo plazo. Sin embargo, con frecuencia está mal documentado [23, 59]. Lwilla y otros[60] mencionó que el DOTS proporcionado por los trabajadores de salud comunitarios era práctico y sostenible porque permitía optimizar el tiempo de algunos trabajadores de la salud para otras tareas, especialmente en un entorno con un gran aumento en la notificación de casos de tuberculosis. Dudley y otros [61] informaron que, a los 6 años, la atención comunitaria contra la tuberculosis se mantuvo a pesar del creciente número de pacientes que requerían atención. Además, durante este tiempo aumentó el número de trabajadores de salud comunitarios. Sin embargo, la replicabilidad de este modelo varía según el entorno comunitario. En entornos urbanizados más complejos, una barrera que se encuentra es que los servicios de salud se administran sin el apoyo de organizaciones comunitarias. Por lo tanto, las intervenciones con participación comunitaria activa dependen de la disponibilidad de estructuras comunitarias organizadas que puedan proporcionar sus recursos y apoyar los sistemas de salud. De manera similar, Wieland et al. [23] realizaron un estudio de caso informando la sostenibilidad de un programa de prevención y control de la tuberculosis (detección de casos) en el centro de educación para adultos del distrito de Escuelas Públicas de Rochester. Informan que esta intervención se ha sostenido durante 8 años gracias al trabajo colaborativo del centro educativo, el departamento de salud pública y la Asociación Comunitaria Saludable de Rochester, cuya misión es promover la salud a través de un enfoque CBPR. Por otro lado, Han et al. [62] mencionan que los programas comunitarios de prevención de la tuberculosis pueden mantenerse en el tiempo cuando se capacita a los trabajadores de salud comunitarios y se forman grupos de movilización social. Teniendo en cuenta esta evidencia, se ha demostrado que la participación comunitaria conduce a cambios significativos para acabar con la tuberculosis. La confianza y el empoderamiento de la comunidad en sus propios recursos y capacidades les permite trabajar de manera diferente para abordar problemas complejos como la tuberculosis.

Por otro lado, con respecto a los estudios de rentabilidad del DOTS proporcionado por los trabajadores de salud comunitarios, los estudios mostraron que el DOTS proporcionado por los trabajadores de salud comunitarios era más rentable y más rentable con el tiempo. Además, los trabajadores de salud comunitarios no sólo brindan tratamiento, sino que también brindan educación primaria de salud, vacunación, seguimiento de grupos de riesgo, apoyo emocional y otros servicios sociales en beneficio de las personas con tuberculosis y sus familias. [27-29] Por lo tanto, el DOTS proporcionado por la comunidad demuestra que es una alternativa que se puede utilizar para los programas para acabar con la tuberculosis.

Por lo tanto, los programas de TB que consideran a la comunidad como una parte importante de sus actividades necesitan establecer una comunicación sostenida (sistema de contacto con voluntarios) y un contacto frecuente con la organización comunitaria. Además, los trabajadores de salud comunitarios deben recibir retroalimentación sobre el progreso del programa, así como participar en cualquier cambio o nueva iniciativa. Además, el empoderamiento de los trabajadores de salud comunitarios les permite formar nuevos voluntarios para llevar a cabo las actividades [6].

**Fortalezas y limitaciones**

Esta revisión del alcance tiene algunas limitaciones. Se identificaron los estudios publicados en inglés (97,69%) y español (2,31%) porque la mayor fuente de evidencia se presenta en esos idiomas. No descartamos la posibilidad de contar con estudios que cumplan con nuestros criterios de elegibilidad y que hayan sido publicados en otros idiomas. Sin embargo, se realizó una búsqueda exhaustiva en bases de datos de salud reconocidas, se realizó una búsqueda de literatura gris y se recopilaron estudios mediante la búsqueda de literatura y el contacto con los autores para obtener más información. Además, no se realizó una evaluación crítica del diseño de los estudios incluidos, lo que limita la evaluación y caracterización de la calidad y certeza de la evidencia que permite visualizar las bases metodológicas de la evidencia actual sobre este tema. Sería importante que esto se evalúe en futuras revisiones sistemáticas. Sin embargo, creemos que nuestro estudio, al sintetizar la evidencia actual sobre intervenciones o estudios en los que la comunidad ha participado activamente en su desarrollo y/o implementación, agrega valor adicional para que los programas locales y locales de prevención y atención de la tuberculosis consideren a la comunidad como un actor fundamental. parte de las estrategias para lograr sus objetivos de erradicar la tuberculosis [63].

**Investigación futura**

Con base en nuestra revisión de alcance, hemos identificado que hay escasez de información de estudios que cumplan con los principios básicos de CBPR en la población afectada por tuberculosis. Además, se necesitan más estudios para evaluar la rentabilidad y la sostenibilidad de las intervenciones de participación comunitaria para acabar con la tuberculosis a largo plazo. Las asociaciones estratégicas de investigación CBPR pueden movilizar y organizar esfuerzos comunitarios a mayor escala para establecer políticas que permitan el cambio de políticas sociales y económicas necesarias para lograr la equidad en salud [14, 64, 65].

**Conclusiones**

No se ha informado de manera consistente sobre la participación comunitaria directa en la prevención y atención de la tuberculosis en investigaciones con un enfoque de investigación participativa basada en la comunidad (CBPR). Sin embargo, según nuestro análisis, los estudios encontrados muestran que la participación activa de la comunidad presenta una tendencia positiva hacia el fortalecimiento de los programas de prevención y atención de la tuberculosis. Además, la participación de miembros clave de la comunidad no sólo fortalece las capacidades de los sistemas de salud para generar estrategias y planes de acción para la prevención y atención de la tuberculosis, sino que también permite una mejor comprensión de la enfermedad desde la perspectiva del paciente por identificar barreras y oportunidades a lo largo de la cascada de atención a los pacientes afectados por la tuberculosis en el "Sur Global". Esta revisión de alcance también permite mostrar que existe la necesidad de realizar estudios con el enfoque CBPR en la población afectada por tuberculosis debido al gran componente social, como lo son los determinantes sociales de la salud, que afecta a esta enfermedad. Esto parece indicar que se requieren estudios que reestructuren el enfoque de una investigación tradicional a uno con enfoque CBPR, donde la población sea considerada como un fin para los objetivos del estudio. La participación activa de las comunidades en la cocreación de soluciones va más allá del ámbito biomédico y ofrece un camino crítico para los gobiernos regionales y locales en la lucha por erradicar la tuberculosis.

**Agradecimientos:** Los autores agradecen todo el apoyo brindado por la Dra. Anna Biszaha, quien brindó capacitación para desarrollar este estudio.

**Material Suplementario**

Archivo S1. PRISMA – ScR lista de chequeo

Archivo S2. Protocolo

Archivo S3. Lista de revistas científicas

Archivo S4. Términos de búsqueda y palabras clave

Archivo S5. Estrategias de búsqueda en cada base de datos y repositorio de información.

**Referencias**

1. Tuberculosis (TB) [Internet] 2023 [cited 2023 March 18]. Available from: https://www.who.int/news-room/fact-sheets/detail/tuberculosis.
2. Pedrazzoli D, Boccia D, Dodd PJ, Lönnroth K, Dowdy DW, Siroka A, et al. Modelling the social and structural determinants of tuberculosis: opportunities and challenges. Int J Tuberc Lung Dis. 2017 Sep 1;21(9):957-964. doi: 10.5588/ijtld.16.0906.
3. Duarte R, Lönnroth K, Carvalho C, Lima F, Carvalho ACC, Muñoz-Torrico M, et al. Tuberculosis, social determinants and co-morbidities (including HIV). Pulmonology. 2018;24(2):115-119. doi: 10.1016/j.rppnen.2017.11.003.
4. Balogun M, Sekoni A, Meloni ST, Odukoya O, Onajole A, Longe-Peters O, et al. Trained community volunteers improve tuberculosis knowledge and attitudes among adults in a periurban community in southwest Nigeria. Am J Trop Med Hyg. 2015;92(3):625-32. doi: 10.4269/ajtmh.14-0527.
5. WHO: Community contribution to TB care: practice and policy. In WHO. Geneva: World Health Organization. 2003 [cited 2023 March 18]. Available from: https://www.comminit.com/global/content/community-contribution-tb-care-practice-and-policy.
6. Arshad A, Salam RA, Lassi ZS, Das JK, Naqvi I, Bhutta ZA. Community based interventions for the prevention and control of tuberculosis. Infect Dis Poverty. 2014;3:27. doi: 10.1186/2049-9957-3-27.
7. Advocacy, communication and social mobilization [Internet]. Implementing the WHO Stop TB Strategy: A Handbook for National Tuberculosis Control Programmes. World Health Organization. 2008 [cited 2023 March 18]. Available from: https://www.ncbi.nlm.nih.gov/books/NBK310747/.
8. WHO Civil Society Task Force on TB: engagement with civil society as a driver for change: progress report [Internet]. 2022 [cited 2023 March 18]. Available from: https://www.who.int/publications-detail-redirect/9789240049765.
9. Global Tuberculosis Report 2022 [Internet]. [cited 2023 March 18]. Available from: https://www.who.int/teams/global-tuberculosis-programme/tb-reports/global-tuberculosis-report-2022.
10. Towards tuberculosis elimination: an action framework in low-incidence countries [Internet]. 2014 [cited 2023 March 18]. Available from: https://www.who.int/publications-detail-redirect/9789241507707.
11. Organización Mundial de la S. Engage-TB: integración de las actividades comunitarias de lucha contra la tuberculosis en el trabajo de las organizaciones no gubernamentales y otras organizaciones de la sociedad civil: manual de aplicación. Ginebra: Organización Mundial de la Salud. 2012 [cited 2023 March 18]. Available from: https://www.who.int/es/publications/i/item/9789241507097
12. Empowering communities to end TB with the ENGAGE-TB approach [Internet]. 2015 [cited 2023 March 18]. Available from: https://www.who.int/publications/i/item/WHO-HTM-TB-2015.27
13. TB Alliance. 2021 [cited 2022 October 16]. Available from: https://www.tballiance.org/about/mission.
14. Harris DA, Pensa MA, Redlich CA, Pisani MA, Rosenthal MS. Community-based Participatory Research Is Needed to Address Pulmonary Health Disparities. Ann Am Thorac Soc. 2016;13(8):1231-8. doi: 10.1513/AnnalsATS.201601-054PS.
15. Brunette MJ, Curioso WH. Sistemas de salud móvil integrados: Rol de los factores socioculturales y el enfoque de sistemas sociotécnico. Rev Peru Med Exp Salud Publica. 2017;34(3):544-50.
16. Israel BA, Schulz AJ, Parker EA, Becker AB. Review of community-based research: assessing partnership approaches to improve public health. Annu Rev Public Health. 1998;19:173-202. doi: 10.1146/annurev.publhealth.19.1.173.
17. Salimi Y, Shahandeh K, Malekafzali H, Loori N, Kheiltash A, Jamshidi E, et al. Is Community-based Participatory Research (CBPR) Useful? A Systematic Review on Papers in a Decade. Int J Prev Med. 2012;3(6):386-93.
18. Musa BM, Iliyasu Z, Yusuf SM, Uloko AE. Systematic review and metanalysis on community based interventions in tuberculosis care in developing countries. Niger J Med. 2014;23(2):103-17.
19. Tricco AC, Lillie E, Zarin W, O'Brien KK, Colquhoun H, Levac D, et al. PRISMA Extension for Scoping Reviews (PRISMA-ScR): Checklist and Explanation. Ann Intern Med. 2018;169(7):467-473. doi: 10.7326/M18-0850.
20. Arksey H, O'Malley L. Scoping studies: Towards a Methodological Framework. Int J Soc Res Methodol. 2005;8:19–32. doi: 10.1080/1364557032000119616.
21. Levac D, Colquhoun H, O'Brien KK. Scoping studies: advancing the methodology. Implement Sci. 2010;5:69. doi: 10.1186/1748-5908-5-69.
22. George AS, Mehra V, Scott K, Sriram V. Community Participation in Health Systems Research: A Systematic Review Assessing the State of Research, the Nature of Interventions Involved and the Features of Engagement with Communities. PLoS One. 2015;10(10):e0141091. doi: 10.1371/journal.pone.0141091.
23. Wieland ML, Nigon JA, Weis JA, Espinda-Brandt L, Beck D, Sia IG. Sustainability of a Tuberculosis Screening Program at an Adult Education Center Through Community-Based Participatory Research. J Public Health Manag Pract. 2019;25(6):602-605. doi: 10.1097/PHH.0000000000000851.
24. Turusbekova N, Popa C, Dragos M, van der Werf MJ, Dinca I. Strengthening TB infection control in specialized health facilities in Romania - using a participatory approach. Public health. 2016;131:75-81. doi: 10.1016/j.puhe.2015.10.031.
25. Wieland ML, Weis JA, Olney MW, Alemán M, Sullivan S, Millington K, et al. Screening for tuberculosis at an adult education center: results of a community-based participatory process. Am J Public Health. 2011;101(7):1264-7. doi: 10.2105/AJPH.2010.300024.
26. Gnanasan S, Ting KN, Wong KT, Mohd Ali S, Muttalif AR, Anderson C. Convergence of tuberculosis and diabetes mellitus: time to individualise pharmaceutical care. Int J Clin Pharm. 2011;33(1):44-52. doi: 10.1007/s11096-010-9452-3.
27. Khan MA, Walley JD, Witter SN, Imran A, Safdar N. Costs and cost-effectiveness of different DOT strategies for the treatment of tuberculosis in Pakistan. Directly Observed Treatment. Health Policy Plan. 2002;17(2):178-86. doi: 10.1093/heapol/17.2.178.
28. Sinanovic E, Floyd K, Dudley L, Azevedo V, Grant R, Maher D. Cost and cost-effectiveness of community-based care for tuberculosis in Cape Town, South Africa. Int J Tuberc Lung Dis. 2003;7(9 Suppl 1):S56-62.
29. Prado TN, Wada N, Guidoni LM, Golub JE, Dietze R, Maciel ELN. Cost-effectiveness of community health worker versus home-based guardians for directly observed treatment of tuberculosis in Vitória, Espírito Santo State, Brazil. Cad Saúde Pública. 2011;27:944-52. doi: [10.1590/S0102-311X2011000500012](https://doi.org/10.1590/S0102-311X2011000500012).
30. World Health Organization. Global tuberculosis report 2022. Available from: https://www.who.int/publications/i/item/9789240061729.
31. Tuberculosis en las Américas. Informe regional 2021. Washington, D.C.: Organización Panamericana de la Salud; 2021. Licencia: CC BY-NC-SA 3.0 IGO. https://doi.org/10.37774/9789275324479.
32. Yassin MA, Datiko DG, Tulloch O, Markos P, Aschalew M, Shargie EB, et al. Innovative community-based approaches doubled tuberculosis case notification and improve treatment outcome in Southern Ethiopia. PLoS One. 2013;8(5):e63174. doi: 10.1371/journal.pone.0063174.
33. Banerjee A, Sharma BV, Ray A, Kannuri NK, Venkateswarlu TV. Acceptability of traditional healers as directly observed treatment providers in tuberculosis control in a tribal area of Andhra Pradesh, India. Int J Tuberc Lung Dis. 2004;8(10):1260-5.
34. Dewi C, Barclay L, Wilson S, Passey M. An asset-based intervention with tuberculosis groups in rural Indonesian villages: Overview and lessons learned. Community Dev J. 2018;53(2):340-57. doi: 10.1093/cdj/bsw037
35. Oshi DC, Omeje JC, Oshi SN, Alobu IN, Chukwu NE, Nwokocha C, et al. An evaluation of innovative community-based approaches and systematic tuberculosis screening to improve tuberculosis case detection in Ebonyi State, Nigeria. Int J Mycobacteriol. 2017;6(3):246-252. doi: 10.4103/ijmy.ijmy_91_17. doi: 10.4103/ijmy.ijmy_91_17.
36. Fox GJ, Loan le P, Nhung NV, Loi NT, Sy DN, Britton WJ, et al. Barriers to adherence with tuberculosis contact investigation in six provinces of Vietnam: a nested case-control study. BMC Infect Dis. 2015;15:103. doi: 10.1186/s12879-015-0816-0.
37. Morishita F, Garfin AM, Lew W, Oh KH, Yadav RP, Reston JC, et al. Bringing state-of-the-art diagnostics to vulnerable populations: The use of a mobile screening unit in active case finding for tuberculosis in Palawan, the Philippines. PLoS One. 2017;12(2):e0171310. doi: 10.1371/journal.pone.0171310.
38. Lorent N, Choun K, Thai S, Kim T, Huy S, Pe R, et al. Community-based active tuberculosis case finding in poor urban settlements of Phnom Penh, Cambodia: a feasible and effective strategy. PLoS One. 2014;9(3):e92754. doi: 10.1371/journal.pone.0092754.
39. Corbett EL, Bandason T, Duong T, Dauya E, Makamure B, Churchyard GJ, et al. Comparison of two active case-finding strategies for community-based diagnosis of symptomatic smear-positive tuberculosis and control of infectious tuberculosis in Harare, Zimbabwe (DETECTB): a cluster-randomised trial. Lancet. 2010;376(9748):1244-53. doi: 10.1016/S0140-6736(10)61425-0.
40. Oshi DC, Chukwu JN, Nwafor CC, Meka AO, Madichie NO, Ogbudebe CL, et al. Does intensified case finding increase tuberculosis case notification among children in resource-poor settings? A report from Nigeria. Int J Mycobacteriol. 2016;5(1):44-50. doi: 10.1016/j.ijmyco.2015.10.007.
41. Colvin C, Mugyabuso J, Munuo G, Lyimo J, Oren E, Mkomwa Z, et al. Evaluation of community-based interventions to improve TB case detection in a rural district of Tanzania. Glob Health Sci Pract. 2014;2(2):219-25. doi: 10.9745/GHSP-D-14-00026.
42. Datiko DG, Yassin MA, Tulloch O, Asnake G, Tesema T, Jamal H, et al. Exploring providers' perspectives of a community based TB approach in Southern Ethiopia: implication for community based approaches. BMC Health Serv Res. 2015;15:501. doi: 10.1186/s12913-015-1149-9.
43. Becx-Bleumink M, Wibowo H, Apriani W, Vrakking H. High tuberculosis notification and treatment success rates through community participation in central Sulawesi, Republic of Indonesia. Int J Tuberc Lung Dis. 2001;5(10):920-5.
44. Boffa J, Mayan M, Ndlovu S, Mhlaba T, Williamson T, Sauve R, et al. The role of agency in the implementation of Isoniazid Preventive Therapy (IPT): Lessons from oMakoti in uMgungundlovu District, South Africa. PloS one. 2018;13(3):e0193571. doi: 10.1371/journal.pone.0193571.
45. Wieland ML, Weis JA, Yawn BP, Sullivan SM, Millington KL, Smith CM, et al. Perceptions of tuberculosis among immigrants and refugees at an adult education center: a community-based participatory research approach. J Immigr Minor Health. 2012;14(1):14-22. doi: 10.1007/s10903-010-9391-z.
46. Wieland ML, Nelson J, Palmer T, O'Hara C, Weis JA, Nigon JA, et al. Evaluation of a tuberculosis education video among immigrants and refugees at an adult education center: a community-based participatory approach. J Health Commun. 2013;18(3):343-53. doi: 10.1080/10810730.2012.727952.
47. Chen E, Leos C, Kowitt SD, Moracco KE. Enhancing Community-Based Participatory Research Through Human-Centered Design Strategies. Health Promot Pract. 2020;21(1):37-48. doi: 10.1177/1524839919850557.
48. Ward M, Schulz AJ, Israel BA, Rice K, Martenies SE, Markarian E. A conceptual framework for evaluating health equity promotion within community-based participatory research partnerships. Eval Program Plann. 2018;70:25-34. doi: 10.1016/j.evalprogplan.2018.04.014.
49. Chávez-Rimache L., Ugarte-Gil, C., Brunette, M. (2022). Empowering community health workers via health equity research training in TB prevention and control. The Union World Conference on Lung Health. 2023 [cited 2023 July 18]. Available from: https://conf2023.theunion.org/
50. Brunette M. Moving the needle on global health equity: a look back from 2030. Arch Environ Occup Health. 2021;76(3):121-122. doi: 10.1080/19338244.2021.1892922.
51. Global Health Workforce Alliance (2010) Integrating Community Health Workers in National Health Workforce Plans. Geneva: WHO. 2010 [cited 2023 March 18]. Available from: https://chwcentral.org/wp-content/uploads/2013/07/Community-Health-Workers-Key-Messages.pdf
52. Zulu JM, Kinsman J, Michelo C, Hurtig AK. Developing the national community health assistant strategy in Zambia: a policy analysis. Health Res Policy Syst. 2013;11:24. doi: 10.1186/1478-4505-11-24.
53. Drabo M, Zerbo R, Berthe A, Ouedrago L, Konfe S, Mugishe E, et al. [Community involvement in tuberculosis care in three rural health districts of Burkina Faso]. Sante publique (Vandoeuvre-les-Nancy, France). 2009;21(5):485-97.
54. Thomas C, Newell JN, Baral SC, Byanjankar L. The contribution of volunteers to a successful community-orientated tuberculosis treatment centre in an urban setting in Nepal: a qualitative assessment of volunteers' roles and motivations. J Health Organ Manag. 2007;21(6):554-72. doi: 10.1108/14777260710834346.
55. Seutloali T, Napoles L, Bam N. Community health workers in Lesotho: Experiences of health promotion activities. Afr J Prim Health Care Fam Med. 2018;10(1):e1-e8. doi: 10.4102/phcfm.v10i1.1558.
56. Reyes MP, Álvarez G. Formación de promotores para la prevención y control de la tuberculosis en la región fronteriza de Chiapas, México. PSM [Internet]. 2014 June [cited 2023 Oct 16] ; 11( 2 ): 130-146. Available from: http://www.scielo.sa.cr/scielo.php?script=sci_arttext&pid=S1659-02012014000200006&lng=en.
57. Moore JE, Mascarenhas A, Bain J, Straus SE. Developing a comprehensive definition of sustainability. Implement Sci. 2017;12(1):110. doi: 10.1186/s13012-017-0637-1.
58. Neta G, Glasgow RE, Carpenter CR, Grimshaw JM, Rabin BA, Fernandez ME, et al. A Framework for Enhancing the Value of Research for Dissemination and Implementation. Am J Public Health. 2015;105(1):49-57. doi: 10.2105/AJPH.2014.302206.
59. Wiltsey S, Kimberly J, Cook N, Calloway A, Castro F, Charns M. The sustainability of new programs and innovations: a review of the empirical literature and recommendations for future research. Implement Sci. 2012;7:17. doi: 10.1186/1748-5908-7-17.
60. Lwilla F, Schellenberg D, Masanja H, Acosta C, Galindo C, Aponte J, et al. Evaluation of efficacy of community-based vs. institutional-based direct observed short-course treatment for the control of tuberculosis in Kilombero district, Tanzania. Trop Med Int Health. 2003;8(3):204-10. doi: 10.1046/j.1365-3156.2003.00999.x.
61. Dudley L, Azevedo V, Grant R, Schoeman JH, Dikweni L, Maher D. Evaluation of community contribution to tuberculosis control in Cape Town, South Africa. Int J Tuberc Lung Dis. 2003;7(9 Suppl 1):S48-55.
62. Han WW, Saw S, Isaakidis P, Khogali M, Reid A, Hoa N, et al. Different challenges, different approaches and related expenditures of community-based tuberculosis activities by international non-governmental organizations in Myanmar. Infect Dis Poverty. 2017;6(1):59. doi: 10.1186/s40249-017-0263-9.
63. Pan American Health Organization. Sustainable development goals (SDG) 3 - Target 3.3 End the transmission of communicable diseases such as HIV, malaria, TB and neglected diseases - PAHO/WHO | Pan American Health Organization [Internet]. 2022 [citado 2022 November 1]. Available from: https://www.paho.org/en/sdg-3-target-3-3?topic=All&d%5Bmin%5D=&d%5Bmax%5D=&page=1.
64. Izumi BT, Schulz AJ, Israel BA, Reyes AG, Martin J, Lichtenstein RL, et al. The one-pager: a practical policy advocacy tool for translating community-based participatory research into action. Prog Community Health Partnersh. 2010;4(2):141-7. doi: 10.1353/cpr.0.0114.
65. Israel BA, Coombe CM, Cheezum RR, Schulz AJ, McGranaghan RJ, Lichtenstein R, et al. Community-based participatory research: a capacity-building approach for policy advocacy aimed at eliminating health disparities. Am J Public Health. 2010;100(11):2094-102. doi: 10.2105/AJPH.2009.170506.
